# An inter-laboratory comparison of urinary 3-hydroxypropylmercapturic acid measurement demonstrates good reproducibility between laboratories

**DOI:** 10.1186/1756-0500-4-391

**Published:** 2011-10-10

**Authors:** Emmanuel Minet, Graham Errington, Gerhard Scherer, Kirk Newland, Mehran Sharifi, Brian Bailey, Mike McEwan, Francis Cheung

**Affiliations:** 1 British American Tobacco, Group Research and Development, Regents Park Road, Southampton, SO15 8TL, UK; 2 Analytisch-Biologisches Forschungslabor GmbH, Goethestrasse 20, 80336 Muenchen, Germany; 3 Celerion, 621 Rose Street, Lincoln, NE 68502, USA; 4 Labstat International Inc., 262 Manitou Drive, Kitchener, Ontario N2C 1L3, Canada; 5 Covance Laboratories Ltd, Otley Road, Harrogate, HG3 1PY, UK

## Abstract

**Background:**

Biomarkers have been used extensively in clinical studies to assess toxicant exposure in smokers and non-smokers and have recently been used in the evaluation of novel tobacco products. The urinary metabolite 3-HPMA, a metabolite of the major tobacco smoke toxicity contributor acrolein, is one example of a biomarker used to measure exposure to tobacco smoke. A number of laboratories have developed liquid chromatography with tandem mass spectrometry (LC-MS/MS) based methods to measure urinary 3-HPMA; however, it is unclear to what extent the data obtained by these different laboratories are comparable.

**Findings:**

This report describes an inter-laboratory comparison carried out to evaluate the comparability of 3-HPMA measurement between four laboratories. A common set of spiked and authentic smoker and non-smoker urine samples were used. Each laboratory used their in-house LC-MS/MS method and a common internal standard. A comparison of the repeatability ('r'), reproducibility ('R'), and coefficient of variation for 3-HPMA demonstrated that within-laboratory variation was consistently lower than between-laboratory variation. The average inter-laboratory coefficient of variation was 7% for fortified urine samples and 16.2% for authentic urine samples. Together, this represents an inter-laboratory variation of 12.2%.

**Conclusion:**

The results from this first inter-laboratory comparison for the measurement of 3-HPMA in urine demonstrate a reasonably good consensus between laboratories. However, some consistent measurement biases were still observed between laboratories, suggesting that additional work may be required to further reduce the inter-laboratory coefficient of variation.

## Background

Cigarette smoke contains thousands of chemicals, including toxicants, which can be categorized as either gases, semi-volatiles (gas/vapor phase), or particles ("tar" phase) [1]. Machine-measured cigarette yields under the ISO testing regimen do not provide an accurate estimate of human exposure to cigarette smoke toxicants [2]. These limitations have led to the development of methods to quantify biomarkers for specific toxicants in biological fluids such as urine, saliva, and plasma [3].

**Figure 1:**
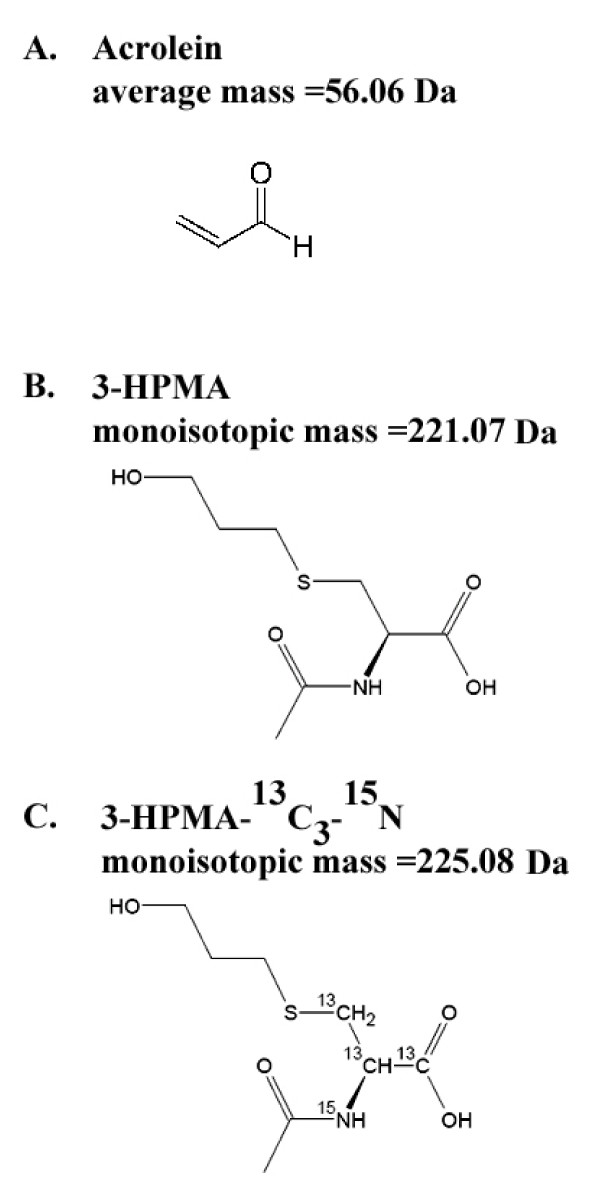
**Chemical structure of acrolein (**A**), 3-HPMA (**B**), and 3-HPMA-^13^C_3_-^15^N (**C**)**. 3-HPMA and 3-HPMA-^13^C_3_-^15^N molecular weights are also indicated.

The gas phase, tobacco smoke toxicant acrolein [CAS:107-02-8] (Figure 1A) has been identified by the World Health Organization (WHO) study group on Tobacco Product Regulation (TobReg) as a major contributor to smoke toxicity [4]. This evaluation was based on the concentration of acrolein in smoke and its toxicity potency factor (cancer and non-cancer), established using various models. 3-hydroxypropylmercapturic acid (3-HPMA) is the major urinary metabolite of acrolein (Figure 1B) [5], and it can be quantified using LC-MS based methods [3,5].

One critical element in the measurement and interpretation of biomarker data (including 3-HPMA) is the comparability in method analysis between different laboratories, which can use different methodologies. For instance, a study conducted by Biber and colleagues on a common set of urinary and plasma samples comparing nicotine and cotinine data from eleven laboratories concluded that individual values could vary significantly between laboratories [6]. In a more recent study, Bernert and colleagues showed that good measurement reproducibility for cotinine in a common set of samples could be achieved between six laboratories, when a standardized HPLC-UV method was used [7].

In this study we tested the reproducibility of 3-HPMA measurement between four laboratories using their in-house method and a common set of fortified and authentic urine samples. Each laboratory used a common reference compound and the internal standard 3-HPMA-^13^C_3_-^15^N.

## Material and methods

### Reagents and samples

Synthetic 3-HPMA (reference compound, Figure 1B) and 3-HPMA-^13^C_3_-^15^N (internal standard, Figure 1C) were obtained from AptoChem (Montreal, Canada). 3-HPMA-^13^C_3_-^15^N was ordered as a custom synthesis and the same lot was used by each laboratory. 3-HPMA-d_3 _was supplied by Toronto Research Chemicals (North York, Canada). Pooled non-smoker urine samples were supplied fortified with 3-HPMA by RECIPE Chemicals (Munich, Germany). Four concentrations of synthetic 3-HPMA were used: unspiked (background ≈30-50 ng/ml), 400 ng/ml, 1200 ng/ml, and 3600 ng/ml 3-HPMA. Prior to distribution, the samples were quantified using one of the participating laboratories (Lab 2) in order to ensure the quality of the preparation. The samples were then portioned into 5-ml-aliquots, lyophilized and shipped to the laboratories in triplicates of each sample (3 × 4 vials with lyophilized urine). The laboratories were advised to reconstitute the samples with 5 ml water.

Five authentic urine samples, covering a 3-HPMA concentration range which reflects typical levels in non-smokers to heavy smokers, were aliquoted in triplicates and sent to the participating laboratories (3 × 5 vials). The lyophilized non-smoker urine samples were supplied by RECIPE^® ^(Munich, Germany), a supplier of samples used for quality assurance testing. The smoker samples were obtained as part of a biomarker study conducted previously by BAT. The corresponding study protocol and informed consent forms were approved by the Ethics Committee of the Bayerische landesarztekammer Munich, Germany (v. 18.02.2008), which contained a provision for revisiting the samples for the purpose of biomarker method development. The clinical study was conducted in accordance with the World Medical Association Declaration of Helsinki (World Medical Association, 2004) and International Conference on Harmonisation (ICH) Guidelines for Good Clinical Practice (GCP) (International Conference on Harmonization, 1996).

### Analytical procedure

The samples were labelled with a code number and randomized prior to distribution. 10 mg of each reference compound and the internal standard were dispatched as a dried powder. Samples were received in one batch by each laboratory in September 2009, and all analyses were completed by November 2009. A summary of the analytical methods is given in Table 1, which is based on the performance of QC samples measured prior to the initiation of the study. All four laboratories used their in-house protocol, which included a solid phase extraction step and analysis by LC-ESI-MS/MS. The methods were validated according to FDA guidelines.

**Table 1:** Analytical method characteristics

*Laboratories*	*1*	*2*	*3*	*4*
*Method*	LC-ESI-MS/MS	LC-ESI-MS/MS	LC-ESI-MS/MS	LC-ESI-MS/MS
*mode*	positive	negative	positive	negative
*SPE (column)*	Phenomenex Strata-X	Isolute ENV+	Waters OASIS	Waters OASIS
*SPE recovery (%)*	74	68	75	78
*HPLC column (make)*	Waters Xterra MS C18	Waters HILIC-Silica	Waters Acquity Phenyl	Thermo BioBasic AX
*HPLC column (size)*	50 × 2.1 mm, 2.5 μm	150 × 2.1 mm, 3 μm	100 × 2.1 mm, 1.7 μm	50 × 3 mm, 5 μm
*Quantifier ion (mass)*	m/z 222 - 163	m/z 220 - 89	m/z 222 - 117	m/z 220 -91
*Qualifier ion (mass)*	m/z 222 - 117	m/z 220 - 91		
*precision intra-day (%)*	1.1 to 5.9	1.4 to 8.6	1.1 to 1.5	1.2 to 6.5
*precision inter-day (%)^a^*	5.1 to 5.3	3.3 to 7	1.7 to 3.9	3.3 to 7.5
*accuracy (%)*	93.2 to 102	83.9 to 102	97.6 to 102	96.8 to 101
*LOD (ng/ml)*	2.21	ND^d^	ND	ND^d^
*LOQ (ng/ml)*	7	25	50^f^	35^f^
*Linearity (ng/ml)*	7 to 5400	25 to 10000	50 to 5000	35 to 5000
*Matrix effect (%)*	ND	7.4 to 17	-1.7 to 19.6	-5.5 to 6.3

^a^Values represent the precision range obtained for low, medium, and high concentrations at the time the methods were developed, except for Lab 1 where only low and high concentrations were tested.

^b^Based on calibration standards.

^c^LOD was an estimate based on spiked water, water being used as the SPE solvent.

^d^Not determined

^e^LOQ was established in spiked water, water being used as SPE solvent.

^f^Matrices used were either diluted non-smoker urine or non-smoker urine with very low 3-HPMA background.

### Statistical analysis

Basic statistical analysis was carried out with MINITAB v15.1. Individual value plots were produced to test inter-laboratory variation of 3-HPMA concentrations. A non-parametric Wilcoxon paired t-test was performed to compare preliminary test analyses conducted with different internal standards. Analysis of covariance was used to compare the analytical methods at the four laboratories using the same internal standard (3-HPMA-^13^C_3_-^15^N). Precision statistics, as defined in ISO 5725-2 [8], were used as a measure of random errors, and expressed as repeatability ('r') and reproducibility ('R'). For the purposes of this study, in which each laboratory used its own method, 'R' refers to inter-laboratory variation.

## Results and discussion

**Figure 2:**
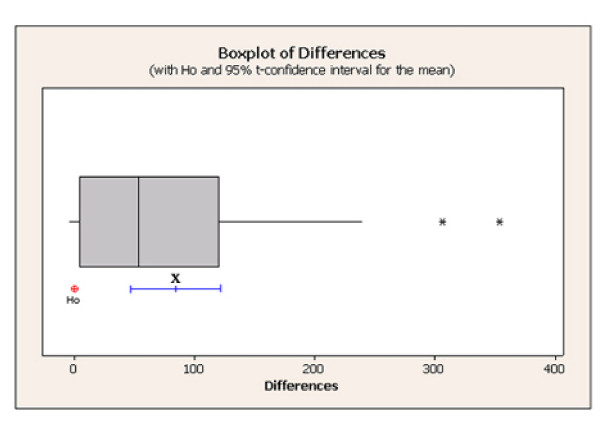
**Boxplot of non-parametric differences between 3-HPMA measured in all samples using 3-HPMA-d_3 _and 3-HPMA-^13^C_3_-^15^N internal standards**. The hypothesis (Ho) is based on no difference (0) between the 3-HPMA-d_3 _measures minus the 3-HPMA-^13^C_3_-^15^N measures. The box plot shows a clear positive difference with p = 0 based on a Wilcoxon paired t-test with a 95% confidence interval for the mean difference (x).

Data for both fortified and authentic urine, using 3-HPMA-^13^C_3_-^15^N as internal standard, were reported by each lab and the corresponding 3-HPMA concentrations (ng/ml) are shown in Table 2. In addition, laboratory 1 repeated the measurements using two different internal standards - 3-HPMA-d_3 _and 3-HPMA-^13^C_3_-^15^N - which were prepared and analyzed on the same day, in order to investigate the potential confounding effects of using different standards under the same analytical conditions. A non-parametric paired t-test showed that the use of 3-HPMA-d_3 _gave consistently higher concentrations than 3-HPMA-^13^C_3_-^15^N (Figure 2), highlighting the importance of standardizing the use of internal standards across each laboratory throughout the study.

**Table 2:** All urinary 3-HPMA data in ng/ml

			*3-HPMA (ng/ml)*			
			
*Samples*			*Lab1*	*Lab2*	*Lab3*	*Lab4*
**1**	**a**	pooled NS^a ^urine	40.6	31.9	< LOQ^b^	< LOQ
	**b**	pooled NS urine	29.8	30.8	< LOQ	< LOQ
	**c**	pooled NS urine	40.0	30.7	< LOQ	< LOQ

**2**	**a**	fortified NS urine	466	492	396	405
	**b**	fortified NS urine	402	470	400	403
	**c**	fortified NS urine	431	471	401	402

**3**	**a**	fortified NS urine	1302	1340	1180	1070
	**b**	fortified NS urine	1230	1270	1160	1140
	**c**	fortified NS urine	1140	1340	1150	1090

**4**	**a**	fortified NS urine	3624	3780	3470	3220
	**b**	fortified NS urine	3714	3820	3420	3210
	**c**	fortified NS urine	3504	3970	3370	3240

**5**	**a**	NS urine	39.8	48.3	< LOQ	< LOQ
	**b**	NS urine	48.2	46.1	< LOQ	36.6
	**c**	NS urine	62.4	46.9	< LOQ	35.1

**6**	**a**	smoker urine	371	376	269	321
	**b**	smoker urine	376	382	294	269
	**c**	smoker urine	371	370	300	258

**7**	**a**	smoker urine	870	842	613	556
	**b**	smoker urine	960	874	673	659
	**c**	smoker urine	840	830	721	619

**8**	**a**	smoker urine	1080	1200	862	969
	**b**	smoker urine	1122	1160	925	914
	**c**	smoker urine	1044	1180	878	929

**9**	**a**	smoker urine	1482	1390	1200	1100
	**b**	smoker urine	1296	1370	1210	1130
	**c**	smoker urine	1260	1390	1100	894

Samples are numbered from 1 to 9. Samples 1 non-smoker urine, samples 2-4 = fortified samples, and samples 5-9 = authentic urine samples. Each sample was aliquoted in triplicates labeled **a**, **b**, and **c**.

^a^NS: non smokers

^b^< LOQ: below limit of quantification

A background level of 40 to 60 ng/ml 3-HPMA was observed in the non-smoker samples selected for this study. This is expected given that acrolein is also the product of lipid peroxidation, fossil fuel combustion, and is found in cooked food [9].

As a quality control check, data from the fortified urine samples (Table 2) were plotted to generate a regression line and the corresponding equation. Using this, the values for the urine samples (Table 2) were recalculated based on the 3-HPMA peak area and the 3-HPMA-^13^C_3_-^15^N internal standard. The calculated concentrations were consistent with the reported concentrations from each lab (Additional file 1).

**Figure 3:**
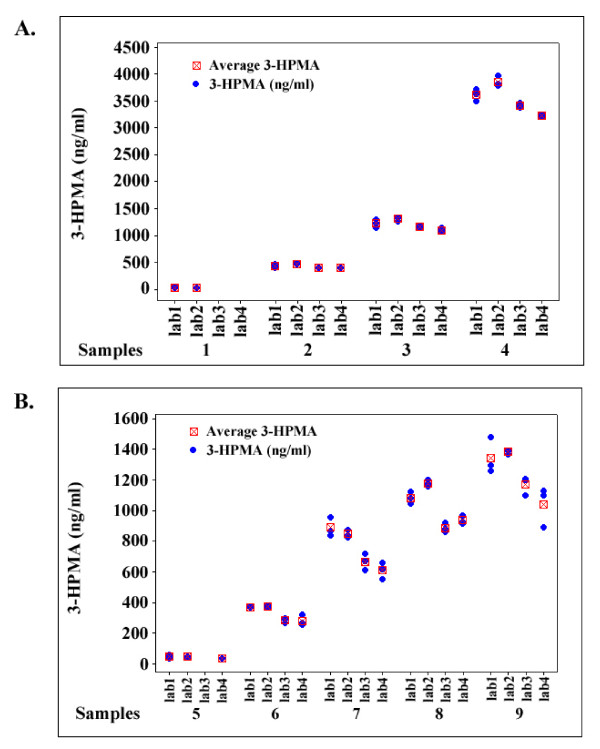
**Individual value plots for 3-HPMA (blue circles) (ng/ml)**. A. Value plot for the fortified samples in four laboratories. B. Value plot for authentic urine samples in four laboratories. Missing values were below the LOQ.

Individual value plots constructed using the sample data indicate a close similarity in the measurements, across the broad range of 3-HPMA concentrations, for all four laboratories using 3-HPMA-^13^C_3_-^15^N (Figure 3). However, an analysis of covariance (ANOVA) indicated a significant variation between laboratories still existed (Table 3). A closer fit could be observed between lab 1 and 2, and between lab 3 and 4. The coefficients of variation, giving an estimate of the imprecision for repeated measures at different concentration ranges, are also reported in Table 3. The imprecision for each concentration range should be interpreted carefully since the replicate measures were obtained from three aliquots from a single solution rather than a triplicate measure of a unique sample. The fortified samples (samples 2 to 4), were used as an internal calibration reference to calculate accuracies (Table 4).

**Table 3:** One way ANOVA for 3-HPMA *vs *laboratories (lab1, 2, 3, and 4) for each set of samples

		*Mean 3-HPMA (ng/ml)*	*StDev*	*CoV*	*P value (one-way Anova)*
**Sample 1**	**lab1**	36.8	6.1	16.5	0.186
	**lab2**	31.13	0.7	2.1	
	**lab3**	< LOQ	NA	NA	
	**lab4**	< LOQ	NA	NA	

**Sample 2**	**lab1**	433	32.2	7.4	0.002
	**lab2**	477.7	12.4	2.6	
	**lab3**	399	2.6	0.7	
	**lab4**	403.3	1.5	0.4	

**Sample 3**	**lab1**	1224	81.2	6.6	0.004
	**lab2**	1316.7	40.4	3.1	
	**lab3**	1163.3	15.3	1.3	
	**lab4**	1100	36.1	3.3	

**Sample 4**	**lab1**	3614	105.4	2.9	0.000
	**lab2**	3856.7	100.2	2.6	
	**lab3**	3420	50	1.5	
	**lab4**	3223.3	15.3	0.5	

**Sample 5**	**lab1**	50.1	11.4	22.8	0.182
	**lab2**	47.1	1.1	2.4	
	**lab3**	< LOQ	NA	NA	
	**lab4**	35.8	1.1	3	

**Sample 6**	**lab1**	372.4	2.77	0.7	0.000
	**lab2**	376	6	1.6	
	**lab3**	287.7	16.4	5.7	
	**lab4**	282.7	33.6	11.9	

**Sample 7**	**lab1**	890	62.4	7	0.000
	**lab2**	848.7	22.7	2.7	
	**lab3**	669	54.1	8.1	
	**lab4**	611.3	51.9	8.5	

**Sample 8**	**lab1**	1082	39	3.6	0.000
	**lab2**	1180	20	1.7	
	**lab3**	888.3	32.7	3.7	
	**lab4**	937.3	28.4	3	

**Sample 9**	**lab1**	1346	119.1	8.85	0.006
	**lab2**	1383.3	11.5	0.8	
	**lab3**	1170	60.8	5.2	
	**lab4**	1041.3	128.5	12.3	

^g^NA: not applicable, fewer than three data points due to at least one measure < LOQ.

**Table 4:** Accuracies calculated for each laboratory based on 3-HPMA fortified samples at 400 ng/ml (samples 2), 1200 ng/ml (samples 3), and 3600 ng/ml (samples 4)

	Accuracies (%)			
***Fortified sample (ng/ml)***	***Lab1***	***Lab2***	***Lab3***	***Lab4***

400 ng/ml	108	119	99	101
1200 ng/ml	102	110	97	92
3600 ng/ml	100	107	95	90

^a^Average calculated over three independent measures

A comparison of the repeatability ('r'), reproducibility ('R'), and coefficient of variation for 3-HPMA demonstrated that within-laboratory variation was consistently lower than between-laboratory variation. The average intra-laboratory CoV was 5%, while the average inter-laboratory CoV was 12.2% (Table 5). The average inter-laboratory coefficient of variation was 7% for the fortified urine samples and 16.2% for the authentic urine samples. These results show close comparability with those observed by Biber and colleagues, where samples spiked with cotinine had an inter-laboratory CoV ranging from 3 to 19%, while a CoV range of 4 to 59% was reported for authentic urine samples of smokers [10].

**Table 5:** Repeatability, reproducibility, and intra, inter-laboratory coefficient of variation for 3-HPMA between the four participating laboratories

*Samples*	*Mean 3-HPMA (ng/ml)^a^*	*r^b^*	*R^c^*	*CoV within (%)^d^*	*CoV between (%)^e^*
**1**	34	5.8	13.9	6.1	NA^f^
**2**	428	50	100.8	4.2	8.4
**3**	1201	142.7	261.8	4.2	7.8
**4**	3528	268.4	460.3	2.7	4.7
**5**	45	7.6	24.2	5.9	NA^f^
**6**	330	54.9	138	6	15
**7**	755	143.3	363.4	6.8	17.2
**8**	1021	89.1	346.4	3.1	12.1
**9**	1235	223.1	710.1	6.5	20.5

^a^Mean of individual 3-HPMA values for all participating laboratories and for the corresponding sample set

^b^Reproducibility

^c^Repeatability

^d^composite intralaboratory coefficient of variation

^e^inter-laboratory coefficient of variation for the four participating laboratories

^f^NA: not applicable due to data < LOQ

The overall average inter-laboratory coefficient of variation for all samples in this study was 12.2%. A CoV value higher than 10% might indicate that there is still some room for improvement; however, this seems to be in line with WHO standardized clinical methods, which in previous studies have reported average inter-laboratory coefficients of variation (CoV) above 10% [11-13].

The results from this first inter-laboratory comparison for the measurement of 3-HPMA in urine demonstrate a reasonably good consensus between laboratories, with an average CoV of 12.2%. However, some consistent measurement biases were still observed between laboratories, suggesting that additional work may be required to reduce the inter-laboratory CoV even further.

## Abbreviations

3-HPMA: 3-hydroxypropylmercapturic acid; CoV: coefficient of variation; ESI: electrospray; LC-MS: liquid chromatography-mass spectrometry; r: repeatability; R: reproducibility; TobReg: tobacco product regulation; UV: ultraviolet; WHO: world health organization

## Competing interests

British American Tobacco (BAT) funded and designed the study.

The participating laboratories are service providers for Biomarkers analysis and have previously conducted biomarkers analyses for BAT.

## Authors' contributions

EM, MM, FC, and GS designed the study. GS, KN, BB, and MS conducted the sample analyses. GE performed the statistical analysis. EM and FC drafted the manuscript. All authors read and approved the final manuscript.

Written informed consent was obtained from the urine donors for publication of this manuscript and accompanying images. A copy of the form was made available for review by the by the Editor-in-Chief of this journal.

## Supplementary Material

Additional file 1 **Authentic urine 3-HPMA concentrations recalculated using a calibration curve derived from the urine samples spiked with synthetic 3-HPMA**. The 3-HPMA fortified non-smokers urine samples were used to establish 3-HPMA calibration curves for each laboratory. The concentration of 3-HPMA in authentic smokers urine was back-calculated based on the calibration curve and peak areas. The values were compared with the values obtained by each lab with their own calibration curve. Click here for file
