# Supplementary material for: An inter-laboratory comparison of urinary 3-hydroxypropylmercapturic acid measurement demonstrates good reproducibility between laboratories
Source: BMC Res Notes. 2011 Oct 10;4:391. doi: 10.1186/1756-0500-4-391 (PMC3208382; doi:10.1186/1756-0500-4-391)
Supplement: Additional file 1 — Authentic urine 3-HPMA concentrations recalculated using a calibration curve derived from the urine samples spiked with synthetic 3-HPMA. The 3-HPMA fortified non-smokers urine samples were used to establish 3-HPMA calibration curves for each laboratory. The concentration of 3-HPMA in authentic smokers urine was back-calculated based on the calibration curve and peak areas. The values were compared with the values obtained by each lab with their own calibration curve. [file 1756-0500-4-391-S1.DOC]

**Additional file 1:**

**Journal:** BMC Research Notes

**Authors:** Emmanuel Minet, Graham Errington, Gerhard Scherer, Kirk Newland, Mehran Sharifi, Brian Bailey, Mike McEwan, Francis Cheung

**Title:** An inter-laboratory comparison of urinary 3-hydroxypropylmercapturic acid measurement demonstrates good reproducibility between laboratories

**Additional file 1**: Calculated concentration using embedded standards and reported concentration for each laboratory.
